# A proposed framework for an appropriate evaluation scheme for microorganisms as novel foods with a health claim in Europe

**DOI:** 10.1186/s12934-015-0229-1

**Published:** 2015-04-09

**Authors:** Sylvie Miquel, Martin Beaumont, Rebeca Martín, Philippe Langella, Véronique Braesco, Muriel Thomas

**Affiliations:** Commensal and Probiotics-Host Interactions Laboratory, UMR1319 Micalis, INRA, AgroParisTech, Domaine de Vilvert, 78350 Jouy en Josas, France; AgroParisTech, UMR 1319 MICALIS, F-78350 Jouy-en-Josas, France; VAB-nutrition, Clermont-Ferrand, France

**Keywords:** Beneficial microbes, Probiotic, Regulation, Safety, Gastro Intestinal (GI) tract

## Abstract

This paper concerns the procedure and the scientific approach to obtain market authorization for a microorganism to be recognized as a novel food with a health claim. Microorganisms that have not been traditionally used during food production in Europe prior to 1997 are considered as novel foods, which should undergo an in-depth characterization and safety assessment before being authorized on the European market. If a novel food bacterium is claimed to provide a beneficial effect on health, these claims must also be investigated before they can be authorized. Some requirements to obtain novel food certification are shared with those required to obtain a health claim. Although regulation exists that deals with these issues for foods in general, bacteria in food raise a specific set of questions that are only minimally addressed in official documentation. We propose a framework and suggest a list of criteria that should be assessed to obtain marketing authorization and health claim for a bacterium in accordance with European health policy.

## Introduction

The introduction of high throughput sequencing, advanced bioinformatics, and specialized *in vitro* and *in vivo* models has improved the understanding of mechanisms underlying the action of probiotics. Probiotics are defined as “live microorganisms which when administered in adequate amounts, confer a health benefit on the host” [1]. For grammatical reasons, this definition has been recently re-worded by an expert panel of the International Scientific Association for Probiotics and Prebiotics (ISAPP) as, “live microorganisms that, when administrated in adequate amounts, confer a health benefit on the host” [2]. Since February 2013, the European Commission no longer allows companies to communicate assumed health benefits of products based solely on probiotic content. Yet, the use of the term “probiotic”, which is not regulated in Europe, implies that the product has a beneficial health effect, and thus this designation should in theory be considered in itself as a health claim [3]. For instance, the Food Safety Authority of Ireland clearly indicates on its website that the “term probiotic is considered to be a health claim”.

Emerging clinical evidence suggest that beneficial bacteria positively influence a wide range of human health issues, especially digestive health [4-7]. Today, most microorganisms marketed as “probiotics” or beneficial bacteria by the food industry belong to the genera *Lactobacillus* and *Bifidobacterium* [8]. However, these genera are sub-dominant in the intestinal microbiota in adults. This observation, in association with rapidly expanding knowledge of the human microbiome, suggests that a large panel of potential new candidates can be isolated from the dominant members of our adult microbiota. The real challenge for translational projects between scientists and industrial partners will be the introduction of new generations of beneficial strains because there is currently a large gap between the bench and the market. It is indeed difficult for all stakeholders, including academic and industrial partners, to agree unanimously on a system of regulation, which may need to be adjusted on a case-by-case basis.

This review focuses on foods and food ingredients consisting of or including live bacteria and does not cover genetically modified microorganisms, which raise other issues. Bacteria with no history of documented safe use in Europe prior to 1997 are classified as novel foods. Requirements that must be fulfilled for such bacteria to be allowed on the market include the accurate characterization of the strain and a solid demonstration of its safety. If a novel food confers a beneficial effect on health, it will become a novel food associated with a health claim after approval by the EFSA and authorization from the European Commission. However, it remains difficult to define experiments that can be judged as reliable, valuable, and pertinent to support the application of novel bacteria. Here, we summarize criteria that are commonly suggested by authorities or in the scientific literature for the characterization of novel bacteria and the assessment of their safety and efficacy. We also present our point of view about the biological relevance and regulatory significance of these criteria and of the experimental methods often proposed to meet them.

### What is a novel food according to the current European food regulation?

**Figure 1:**
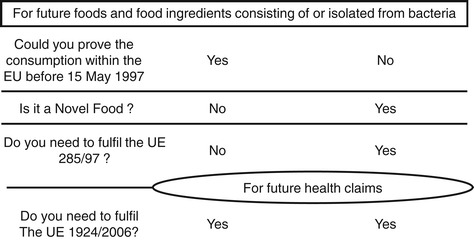
**Statutory text.**

Many microorganisms that are used as food ingredients or food additives have a long history of safe use in food fermentations [8]. However, microorganisms that were not traditionally used in food production in Europe before 1997 are classified as a novel food class 2.2 (see Table 1 and Figure 1). The market and the legislation of novel foods are regulated by the UE 97/618/EC recommendation and regulation N^o^ 258/97 (Table 1 and Figure 1). The launch a Novel Food in Europe is authorized if the product has been thoroughly characterized and its safety has been proven (Figure 1).

**Table 1:** **Statutory text, official documents of the EFSA**

**Novel Food**	
**Recommendation 97/618/EC**	Related to the scientific aspects and the presentation of information necessary to support applications to place novel foods and novel food ingredients on the market
**Regulation N° 258/97**	Related to novel foods and novel food ingredients of the European Parliament and of the European Council
**Novel Food Class 2**	Complex Novel Food from non-Genetically Modified sources according to Categories of novel foods and novel food ingredients identified in Regulation (EC) No 258/97. Intact plants, animals and microorganisms used as foods as well as food components (e.g. complex carbohydrates, fats, proteins or those substances collectively described as dietary fiber) are included. Two sub-classes can be identified:
	2.1 the source of the NF has a history of use in food in the Community.
	2.2 the source of the NF has no history of use in food in the Community.
**Health claim**	
**Regulation N° 1924/2006**	Related to nutrition and health claims and establishes rules governing the Community authorization of health claims should only be authorized in the Community after a scientific assessment of the highest possible standard, to be carried out by the EFSA.

For a novel food to be allowed on the market, those submitting the application are required to do the following:

- # Characterize the microorganism and its metabolites
- # Characterize how the process of production may modify the microorganism
- # Give the history of production and consumption of the microorganism
- # Anticipate the intake/extent of the use and the consumption of the novel food
- # Provide the nutritional composition of the novel food
- # Assess the safety of the microorganism
- # Give toxicological information

An example of an application for a novel food is the public version of an application for the use of *Clostridium butyricum* (CBM588), (which was isolated from a soil sample), as a novel food supplement in the European Union (EU) (Miya-Pro; Public version – *Clostridium butyricum* 588 novel food application).

### What is a novel food with a health claim according to the current European food regulation?

**Figure 2:**
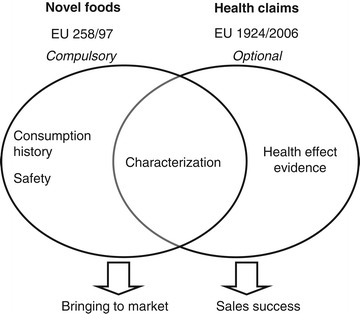
**Novel food or health claim?**

A novel food is not required to have any beneficial nutritional or health-related effects upon consumption. However, a health claim would highlight the benefits of the novel food for the consumer and may help its commercial success (Figure 2). The process for substantiating a health claim for a novel food is the same as for a conventional food. Beneficial nutritional or health claims may be communicated to the consumer only after authorization from the European Commission, which requires a favorable opinion from the EFSA according to EU regulation 1924/2006 (see Table **1** and Figure 1). As shown in Figure 2, some requirements to obtain novel food certification are the same as those required to certify a health claim (e.g. strain characterization).

Overall, most bacteria that will be used in foods for human consumption in the near future will need to comply with two different regulations (EC 258/97 and EC 1924/2006), which largely involve scientific requirements. Successful market applications will require various skills allying the academic and industrial worlds, to address the numerous regulatory, economic, and scientific challenges.

### How can current regulation be improved to ensure the correct characterization of future novel-food microorganisms with a health claim?

Characterization of the strain of microorganism is the first requirement both to apply for novel food marketing authorization and to submit a health claim request (Figure 2). Functional effects are only related to the particular strain and cannot be extended to the rest of the species to which the strain belongs [9,10]. However, the only approved health claim is for yoghurt cultures, which are not defined at the strain level [11]. Thus, if there is a reasonable scientific basis to justify a health benefit for an entire species, then the health claim need not be limited to one strain. Moreover, the ISAPP recently proposed the creation of a general category of probiotics defined at the species level and associated with core benefits [2]. It is also important to consider characterizing the strain in the various environments that it may encounter from the start of the production process until the final consumption of the product.

The EFSA recommends that both the species (DNA-DNA hybridization or 16S rRNA gene sequence analysis) and strain (genetic typing molecular methods) be identified [12]. Indeed, it is essential to identify clearly the species and strain of bacteria based on phenotypic and genotypic data [13] from various methods considered as reliable references in the literature (Table 2) [14]. Although all methods listed in Table 2 are relevant, some may be replaced by whole genome sequencing technologies, which are undergoing rapid development. In addition to characterizing the strain, the sequence may reveal the pathogenic potential of the strain and identify virulence genes; thus, this information may be relevant for the toxicological assessment required for a novel food. Additionally, genomic data are useful to search for functional relationships between genetic elements and the mechanisms underlying probiotic action [15]. Putative proteins and metabolic pathways can also be predicted from genomic sequences and these predictions can be confirmed by metabolomic and transcriptomic profiling with high-throughput techniques. In light of the development of many “omic” techniques, it would be interesting to establish a strain-specific map of genomic and functional interactions. In the near future, it is probable that most bacteria that will be used for the first time in foods for human consumption will have to be entirely sequenced.

**Table 2:** **Criteria and methods often proposed to characterize a strain (required for both novel food and health claim regulations)**

**Taxonomic level**	**Biological assignment**	**Methods**	**Propositions for improvement**
Species	Phenotypic	**GRAM staining**	
		**Morphologic description**	The physiology of the bacterium in environments from production to consumption should be extensively characterized
		**Analytical profile identification (API)**	
	Genotypic	**16S rRNA sequencing** [15,33]	Full genome sequencing should be systematically provided
		**MLST (Multi Locus Sequence Typing)**	
Strain	Metabolic	**Analytical profile identification (API)**	
	Genotypic	**Genome sequencing** [15]: Identification of virulence genes	
		**Pulsed field gel electrophoresis (PFGE)** [20]: evaluation of genetic stability [58]	

Clearly, whole genome sequencing is the gold standard for characterization; however, we can only make sense of these data if we consider a strain in its physiological context and take into account its overall biology. Life cycle, industrial processes, storage history and ingestion may affect the phenotypic properties of bacteria and the potential activities of a particular strain [16]. It could be worthwhile to describe the properties of microorganisms during different physiological states (latency, multiplication, and stationary phases) but also in the context of the various micro-environments that the product could encounter. However, the exact nature of such tests cannot be generalized and specific tests are needed for each microorganism used. For instance, the yoghurt bacteria, *Streptococcus thermophilus* and *Lactobacillus delbrueckii ssp. bulgaricus* present different growth rates and metabolic activities depending on the culture media (milk, presence of lactose or rich media) [17-19]. Although the environment may influence the metabolic activities and/or functions of microorganisms the inverse is also true: microorganisms may affect the physico-chemical conditions of their environment. Moreover, strains could evolve and functional mutations could appear. Thus, pulsed field gel electrophoresis (PFGE) may be informative because a high rate of mutations may lead to the appearance of new characteristics [20]. This is mainly a typing technology, yielding limited data on functionality. Most (functionally-important) SNPs are actually not detected by rare cutting restriction enzyme analysis. Thus, novel-food bacteria should also be highly characterized in the various environments that they will encounter from production to consumption.

The final product of the food matrix in which the microorganism is present should also be precisely characterized:

- to assure the absence of microbial contaminants [21];
- to define the composition of the food because it may affect strain growth;
- to assure proper labeling of the food with regard to macro-nutrients, calories and allergens, especially for particular consumers (e.g. diabetics) [22];
- to define clearly the conditions of use (the target population, the storage conditions and any precautions about the time and regularity of intake) and to support its claimed effect [23].

Concerning intake, it is strongly recommended that the final products contain an adequate amount of live bacteria to provide a health benefit [24,25]. A daily intake of at least 10^8^–10^9^ viable cells, which may be achieved by the daily consumption of at least 100 g of final product, has been suggested as the minimum intake to provide an effect [24].

### How should the safety of microorganisms and their metabolites be assessed? Some suggestions to improve current regulatory requirements

In the EU, the *a priori* safety of some microorganisms is accepted if they benefit from Qualified Presumption of Safety (QPS) status [26].

Most of these microorganisms are Gram positive, non-sporulating or lactic acid bacteria [24]. Lactic acid bacteria, mainly *Bifidobacterium*, *Lactobacillus* and *Streptococcus* are found in many food products and are not dominant in the intestinal microbiota in adults [8,22,27-29]. If the microorganism is not recognized as QPS, a complete assessment of its safety must be carried out according to regulatory requirements. Some adverse side effects that must be monitored are the production of host-deleterious metabolites, systemic infection, inappropriate immune responses, antibiotic (AB) resistance and gene transfer [30]. However, this list is not exhaustive and there is currently no official document summarizing these criteria. Some requirements are mentioned in the scientific literature [21,31,32] and in the Guidelines for the Evaluation of Probiotics in Food published in 2002 by the FAO/WHO working group [13]. However, the biological relevance of these requirements remains a subject of debate. We have ordered and evaluated the criteria that are often proposed to demonstrate the safety of microorganisms into five classes: survival and/or viability along the Gastro Intestinal (GI) tract, effect on intestinal homeostasis, adhesion, metabolic activities, and remote effects (Table 3).

**Table 3:** **Common criteria generally considered as essential for the safety of NF/probiotic products (required for both novel food and health claim regulations)**

	**What**		**How**	**Why**	**Comments and Propositions for improvement**
Survival in GI tract conditions	Resistance to intestinal stress		*In vitro* Growth curves/Detection in feces after consumption	Resistance to GI tract conditions may favor the beneficial effects	Not valuable for all beneficial effects
					Development of new protectors/encapsulators
	Bile salt deconjugation		High-performance liquid chromatography	Large amounts of deconjugated bile salts may have undesirable effects on the human host	Evaluation of property *in vitro* has poor relevance; assessment of bile salt deconjugation *in vivo*
			Mass spectrometry		
Preservation of the homeostasis of gut barrier components	Microbiota	Perturbation of commensal consortium	*In vitro* production of bacteriocins or antibiotics (AB)	Bacteriocins and AB may perturb microbiota.	Development of growth inhibitory references with major commensal bacteria
				AB may interact with a patient’s treatment	
		Antibiotics (AB) resistance	*In silico* *prediction and *in vitro* antibiogram	AB resistance may be transmitted between bacteria	Development of *in vivo* assessment (animal model) indicating the microbiota homeostasis (composition and activities) after probiotic consumption
			Minimal inhibitory concentration test (MIC)		
			Minimal bactericidal concentration test (CMB)		If plasmids are detected: the presence/absence of genes encoding the most common resistance determinants should be characterized
		Presence of plasmids	*In silico* *prediction or DNA extraction followed by analysis by gel electrophoresis	Plasmids favor the transmission of antibiotic resistance	Requirement to up-date the antibiotic list
	Mucus	Mucus degradation	Mucin degradation test (agarose gel or liquid culture)	Excessive mucus degradation may lead to intestinal barrier weakening	The capacity to degrade mucus seems to be a poor criterion to estimate the protective or deleterious effect of bacteria on the intestinal barrier
Adhesion and translocation risk	Intestinal/Mucosal adhesion		Test bacterial strain adhesion to epithelial cell line	Mucosal adhesion may interfere with pathogenic microorganisms, stimulate beneficial cellular processes, or favor bacterial translocation	The intestinal/mucosal adhesion capacity can be either a beneficial or a deleterious criterion
	Intestinal mucosa degradation		Gelatinase activity assay	Mucosal degradation may weaken the intestinal barrier	It could be useful to evaluate *in vivo* translocation capacities
	Hemolytic activity		Blood agar culture	Hemolysis damages red blood cells	
Metabolic activities	D-Lactate production		Colorimetric assay	D-Lactate accumulation in blood leads to acidosis	The production of D-lactate should be compared with the amount produced by usual strains (like in yoghurt)
	Toxin production		Protocol recommended by the European scientific committee of animal nutrition.	Toxic molecules	Establishment of threshold values relevant in humans
	Biogenic amine production		Colorimetric assay	Immune responses such as allergic responses	
Remote effects	Platelet aggregation		Aggregation test	Risk of thrombosis	Development of *ex vivo* protocols (explants, organoids)
	Genotoxicity		Toxicity testing on animals models (chronic and subchronic tests)	Risk of cancer	
	Allergenicity			Allergic response	

(*: if the complete genome is available).

#### Survival and /or viability in the intestinal tract

It is commonly accepted that bacteria must be able to survive in at least the upper part of the GI tract to have a beneficial effect on the host (except bacteria used to improve microbial composition in the oral cavity). An intestinal strain would have to resist extreme GI tract conditions: an intestinal pH gradient (from 4.0 in stomach to 7.0 in the lower part of intestine), bile salts, and pancreatic secretions [33]. All these variables can be monitored *in vitro* by growth curves, although such assays do not totally reflect *in vivo* conditions (Table 3). The amount of bacteria *in vivo* can also be determined by PCR or by isolating and culturing them from stools a few days after ingestion [34]. However, it is debatable whether a bacterium must survive and proliferate along the GI tract to have an effect. For example, polysaccharide A of *Bacteroides fragilis* protects animals from colitis induced by *Helicobacter hepaticus* [35] and the peptidoglycan of the *Lactobacillus salivarius* strain Ls33 also protects against experimentally-induced colitis [36]. Thus, proliferation of the bacterium at the target site is not absolutely required for its effect and a daily consumption of product may suffice. Nonetheless, survival in the GI tract needs to be examined in each particular case, and it would be informative to establish a sensitivity profile of each strain towards pH, bile salts, and pancreatic secretions. Innovative technologies, based for example on encapsulation, are currently being developed to control better the viability of microbes during storage, processing, and in the GI tract [37].

The deconjugation of bile salts is a property that allows bacteria to survive in the GI tract and is implicated in lowering circulating cholesterol levels [38]; however, in excess, this process may be deleterious for the host [39]. Thus, it has been proposed that the bile salt deconjugating activity of probiotics should be evaluated [40], because it could influence bacterial survival and host health. Evaluation of this property (Table 3) *in vitro* may provide little relevant information for the situation *in vivo* because of its complexity, raising the need, as a second step, to perform experiments with an animal model (mono-associated or with a complete microbiota). It is yet not completely clear whether bile salt deconjugating activity is a desirable trait in a novel food/probiotic bacterium. In our opinion, the capacity to deconjugate bile salts is informative when choosing a strain and should therefore be considered.

#### Preservation of intestinal homeostasis by maintaining the integrity of two barrier components of the gut: microbiota and mucus

A key safety requirement is that the use of a new novel food should not perturb the population commensal organisms. It may be useful to examine whether novel food strains inhibit the growth of commensal bacteria, to assess whether and how homeostasis is maintained. This approach requires the assessment *in vitro* of the production of bacteriocins or antibiotics (AB). It would be useful to test *in vitro* if probiotics have bactericidal effects on the main groups of commensal bacteria such as *Bacteroidetes* and *Clostridii*, although this is not required by authorities and is rarely proposed in the literature. Then, the effect of the probiotic consumption on microbiota composition could be assessed with *in vivo* experiments measuring the global metabolic activities of microbiota (such as the production short-chain fatty acids).

Novel food strains should not be able to transmit antibiotic resistance genes to bacteria in their environment to avoid the acquisition and spread of multiple antibiotic resistance [33]. The transmission potential of resistance genes depends on their genetic support (plasmids or chromosome). Unlike resistance genes carried by plasmids, those carried by chromosomes have a much lower risk of transfer. Thus, it is important to consider the genomic location of an antibiotic resistance gene when testing a strain for antibiotic resistance [32]. A list of antibiotics which should be tested to assess antibiotic resistance has been proposed by the EFSA [41]. This list should be updated according to current treatments and the target population (Table 3).

It is also widely reported that bacteria are beneficial if they do not degrade mucus. The rationale is that degrading mucus weakens the intestinal barrier and consequently destabilizes the protective function of the epithelium [42]. However, some commensal bacteria use mucins, the major constituents of mucus, as an energy source and can stimulate host mucus production [43]. Thus, such adaptive cross-talk does not necessarily impair host defense mechanisms. Moreover, mucin degradation tests (agarose gel or liquid culture) often use synthetic mucins from pig gastric mucus. This experimental setting does not accurately represent the typical features of human intestinal mucus, and moreover, the diversity of available energetic substrates *in vivo* is much higher than *in vitro.* Thus, bacteria that degrade mucus *in vitro* (where mucus is the only energy source in culture medium) may not necessarily use this substrate *in vivo*. In our opinion, the capacity to degrade mucus, as assessed by *in vitro* assays, is not a relevant criterion to estimate the protective or deleterious effect of bacteria on the intestinal barrier (Table 3). For instance, administration of living *Akkermansia muciniphila,* which is a mucin-degrading commensal, reverses dietary induced metabolic disorders [44].

#### Adhesion properties and translocation risk

Adhesion to the mucosal layer is commonly mentioned as a factor favoring durable implantation and a highly effective probiotic [13]. This suggests that transient persistence and/or long-term colonization is associated with beneficial mechanisms such as interfering with the growth of pathogenic or potentially pathogenic microorganisms in the body or stimulating other potentially beneficial cellular processes [45]. In our opinion, this assumption is questionable, because proximity to the intestinal mucosa and a long transit time in the gut are not sufficient to maximize the beneficial effects of a strain (Table 3). Indeed, although bacteria are rapidly eliminated, they may act transitorily [46]. For instance, the immune system of germ-free mice can be stimulated by temporary bacterial colonization [47]. These results imply that some microorganisms do not have to colonize permanently the microbiota to affect host responses [46]. In addition, *in vitro* tests have many limits, because adhesion properties and mucus production depend on the cell line under study [48]. We believe that although adhesion is an important characteristic of a strain, it should not be a criterion to estimate the potential effect within the gut. However, *in vitro* tests that assess adhesion also reveal the cytotoxicity of the bacterium on targeted cells. It may be relevant to estimate bacterial translocation because oral treatments containing a high dose of probiotic may be deleterious, especially in immunodeficient patients [49].

#### Metabolic activities: threshold and physiological state matter

It is well known that some bacterial metabolites have deleterious effects; however, the current recommendations provided by authorities are unclear and no maximal threshold for these metabolites has been proposed (Table 3). For instance, bacteria-induced D-lactate production can be viewed as harmful because the accumulation of this metabolite in blood may be neurotoxic and may lead to acidosis [50]. Individuals the most at risk are those with short-bowel syndrome (which results from resection of the small intestine) because of the accumulation D-lactate in their feces [51]. In healthy adults, lactate is not detectable in fecal samples because lactobacilli (main producers of D-lactate) represent a minor group in the microbiota and lactate is degraded by other major bacterial groups [52]. Some strains of *L. delbrueckii* subsp. *bulgaricus* that are widely used in yoghurt fermentation produce D-Lactate [17]. The subsequent risk of acidosis related to D-lactate after the ingestion of yoghurt is not a health threat in healthy individuals [53]. Nearly all lactic acid bacteria, even those widely used for food applications, produce the D-lactate isoform. Moreover, many commensal strains present in the lower GI tract consume D-lactate resulting in cross-feeding that may help to explain the reported butyrogenic effect of certain dietary substrates [52]. Thus, the production of D-lactate is not a risk for healthy people. It remains informative to determine the level of D-lactate production for each microorganism, but the production of D-lactate should not be considered systematically as a metabolic disadvantage for future health-related strains. It could be interesting to compare the amount of D-lactate produced by the considered strains with that produced by *Lactobacilli* currently present in yoghurt.

Lack of toxin or biogenic amine production is essential to ensure strain safety but experimental settings and threshold values are still poorly defined for human applications. The EFSA recommends a protocol originally established for animal nutrition and limited to the use of *Bacillus* species [54]. This may be too specific to be extrapolated to all strains used in human nutrition or health. A relevant alternative strategy may be the use of commensal bacteria as references (Table 3).

Two other tests are frequently mentioned: strain hemolytic activity and platelet aggregation ability. Both are typical features of pathogenic bacteria (Table 3). However, these deleterious effects may only happen if the ingested bacteria end up in the blood. This is an unlikely situation which requires bacterial translocation across a weakened intestinal barrier, which would also favor the translocation of all commensal bacteria. These tests however provide important information about strain pathogenicity [55-57].

#### Remote effects: toxicology testing

It is necessary to evaluate the toxicological profile, including genotoxicity, to establish the safety of a new product [58]. The harmful effects of a particular substance or microorganism are examined, evaluated, and interpreted by testing it on animal models (Table 3). These results are then extrapolated to determine the quantity that will produce similar effects in humans. However, current ethics statements encourage a reduction in the numbers of animals that are used in these tests. Thus, it may be useful to develop new techniques based on viable *ex vivo* tissue explants or organoids (from experimental rodents or humans) that could be co-incubated with bacteria of interest or their respective metabolites [59,60]. In our opinion, *ex vivo* intestinal cultures co-incubated with bacteria or their culture supernatant are promising approaches that should be encouraged to evaluate immune responses (cytokine release in the supernatants and the expression level of immune receptors), epithelium homeostasis (proliferation, differentiation and/or apoptosis) and toxicity markers (e.g. genotoxicity) of new products.

Overall, although *in vitro* testing can be very informative as a first step, the properties of candidate probiotics should always also be assessed *in vivo* because they are strongly influenced by the intestinal ecosystem, the complexity of which cannot be entirely reproduced *in vitro* (biochemical properties, interaction with commensal microorganisms or with the host and substrate availability).

### Are health claims based on a cause-effect relationship between the intake of a microorganism and a benefit to human health?

Clearly, the safety of a microorganism is essential for the authorization of a novel food. However, convincing evidence demonstrating the beneficial effects of a particular microorganism should also be submitted for scientific evaluation to the Nutrition panel of the EFSA. Numerous general and specific guidelines (depending on the health claim) have been published by the EFSA and should be consulted and followed [23,61-63]. Health effects are demonstrated in the same way regardless of the nature of the active component of the novel food (microorganism, nutrient, or any other ingredient). Successful applications preferably involve studies of high methodological and statistical quality examining a well-defined health benefit that can be unambiguously evaluated through changes in a recognized biomarker in humans.

It is particularly difficult to define the beneficial effects after consumption of a bacterium. The ISAPP considers that beneficial microbes that support a healthy digestive tract and a healthy immune system are associated with common general benefits to human health [2]. Indeed, many of the most promising benefits of bacteria involve their effect on the gut, and their interactions with either the gut microbiota or the cells of the intestinal mucosa, especially epithelial and immune cells. These aspects have been extensively studied, and although knowledge is rapidly expanding, the complexity of these interactions is also becoming increasingly apparent. This has prevented clear conclusions from being made about the appropriate markers to examine the effects of a food or a microorganism on human health. As a result, a gap exists between productive research and the effective implementation of these findings in the life of the consumer. This is a good illustration of the challenges of translational research. In addition to research on probiotics and their effects, there is also a strong and sustainable need to document markers and to assess the clinical significance of probiotics on human health. Hopefully, probiotics have many potential activities and although efforts should focus on the microbiota and immune system, other effects that are clearly beneficial to humans, can be more readily assessed. A non-exhaustive list of potential benefits includes reduction in cholesterol levels, decrease of intestinal pain, favored intestinal transit, and reinforcement of the intestinal epithelium.

### The intestinal microbiota: a potential source of novel foods with a health claim

The intestinal microbiota, including commensal bacteria and probiotics, plays a fundamental role in the development and maintenance of intestinal homeostasis by participating in the immune and digestive functions of the GI tract [33]. This homeostasis is crucial for the host health and if disrupted, it may lead to an inappropriate reaction of the immune and digestive systems.

Newly discovered intestinal bacteria may be used for the development of new novel foods containing microorganisms with a health claim [2]. Over the past 20 years, intensive research has led to the in depth characterization of intestinal communities, particularly as a function of age, health status, geographic location, nutritional habits, medical care, and genetic predisposition of the host [64]. It is now widely accepted that intestinal commensal microorganisms participate in the physiology and the health of their host through metabolic, protective, and trophic functions [65]. Host physiology, gut maturation, innate and acquired immune responses and metabolism are largely influenced by the metabolic properties of the microbiota [66-69]. The activity and composition of the microbiota are modulated by external factors, such as diet, making it a highly “malleable” tissue in humans [70]. It was recently proposed that *Faecalibacterium prausnitzii*, which is a major constituent of the intestinal human microbiota, may have prophylactic or therapeutic applications in human health [71,72]. In particular, *F. prausnitzii* is depleted in many intestinal disorders and displays beneficial anti-inflammatory effects on host, suggesting that it may be used to counterbalance the dysbiosis linked to certain diseases [73,74]. Thus, the characterization of several microbial communities from our environment (particularly those of our microbiota) may help to identify new bacterial species with beneficial effects on human health. Interestingly, the recent description of the intestinal metagenome (i.e. all genomes of the bacterial populations present in the intestine), by sequencing strategies has confirmed that the microbial richness of the human gut microbiome correlates with metabolic markers [75]. Therefore, besides being a huge reservoir of unexploited commensal bacteria, our microbiota also has metabolic capacities that are potentially beneficial to human health. We can speculate that this large panel of commensal organisms contains new promising candidates that may be very efficient in the digestive tract because they will be reintroduced into their endogenous ecological niche. However, although the GI tract is their natural niche, most potentially commensal probiotic bacteria have never been used in food products; thus, manufacturers would have to apply for marketing authorization according to the regulation of novel foods.

## Conclusion

We propose a framework that will help academic and industrial communities to explore the potential of bacteria as novel foods with health claims in accordance with European health and nutrition policy. New research on the human microbiome will facilitate the development of mechanistically-driven probiotics. This approach clearly offers a new strategy that may benefit the health of the general population and that of patients with limited therapeutic options; for example, it may provide an alternative to long-term antibiotic use. Further insight into the precise mechanisms of action of new probiotic strains may lead to the development of second generation probiotics with characterized beneficial effects. Until now, yoghurt is the only probiotic food with a health claim [11]. It remains unclear whether these regulations limit or boost creativity and innovation [76]. It is in the interest of stakeholders that this translational subject, at the cross roads of scientific, industrial, and clinical research, is clarified by appropriate regulations [77]. These regulations clearly indicate, to companies as well as to risk assessors and managers, that claims should be based only on very strong scientific evidence. Moreover, it seems necessary to have some flexibility regarding individual studies depending on the particular microorganism involved, the claim area, the target population, and the condition of use. In this “point of view” paper, we have discussed some of the tests proposed for the development of intestinal probiotics, bearing in mind that innovative strategies should be encouraged.
